# Antibiotic Mechanisms and Resistance: Molecular Insights and Therapeutic Strategies

**DOI:** 10.3390/antibiotics15040351

**Published:** 2026-03-29

**Authors:** Haodi Ma, Liying Zhang, Lulu Wang, Zimeng Yang, Junfeng Liu, Haoyang Sun, Shuai Ge, Chunshan Quan

**Affiliations:** 1 Key Laboratory of Biotechnology and Bioresources Utilization (Ministry of Education), College of Life Science, Dalian Minzu University, Dalian 116600, China; 2 Department of Bioengineering, College of Life Science, Dalian Minzu University, Dalian 116600, China

**Keywords:** antibiotic mechanisms, antimicrobial resistance, molecular targets, drug discovery, precision medicine, combination therapy

## Abstract

Antibiotic resistance is a critical global health threat, already causing over 1.27 million deaths annually and projected to exceed 10 million by 2050. This crisis is compounded by stagnation in novel antibiotic discovery, highlighting the need for mechanism–based innovation. Here, we provide an integrative framework linking antibiotic mechanisms of action, bacterial resistance pathways, and emerging therapeutic strategies. Antibiotics are systematically categorized by their molecular targets, cell wall synthesis, membrane integrity, nucleic acid replication, protein synthesis, and metabolic pathways, while resistance mechanisms are outlined in parallel, including enzymatic degradation, target modification, efflux, and permeability barriers. We further highlight novel approaches such as structure–guided drug design, synergistic combinations, nanoparticle delivery, and artificial intelligence–driven discovery. Precision medicine and microbiome modulation are also emphasized as next–generation interventions. By bridging molecular mechanisms with translational strategies, this review outlines opportunities to guide antibiotic innovation and advance precision therapies against the escalating threat of antimicrobial resistance.

## 1. Introduction

Since the discovery of penicillin in 1928, antibiotics have revolutionized modern medicine, dramatically reducing mortality from infectious diseases and enabling complex medical procedures such as organ transplantation and cancer chemotherapy [1,2]. However, these advances are increasingly undermined by the global rise of antimicrobial resistance (AMR), largely fueled by antibiotic misuse and overuse. According to the World Health Organization’s Global Antimicrobial Resistance and Use Surveillance System (GLASS), the median prevalence of *Escherichia coli* resistant to third–generation cephalosporins and methicillin–resistant *Staphylococcus aureus* (MRSA) has reached 42% and 35%, respectively, highlighting a sharp decline in the effectiveness of first–line antibiotics [3]. The WHO now ranks AMR among the greatest public health threats of the 21st century.

Despite decades of research, the development of new antibiotic classes has stagnated, and very few novel mechanisms have been clinically validated since the 1970s [4]. This stagnation stems from multiple factors, including the rapid evolution of resistant bacterial strains, the high cost and long timelines of antibiotic development, and the relatively low financial incentives compared to drugs for chronic diseases. Alarmingly, recent studies show that Gram–negative ESKAPE pathogens can acquire clinically relevant resistance to 13 newly developed antibiotics within just 60 days of in vitro exposure [5], underscoring how quickly bacterial evolution can outpace innovation.

Breaking this cycle requires deeper mechanistic insights into how antibiotics interact with their bacterial targets. While many antibiotics are categorized based on their known targets, such as the cell wall, membrane, or nucleic acid synthesis, these designations rarely capture the complexity of drug target interactions, their physiological consequences, or their links to resistance evolution [6,7]. Moreover, the mechanisms of action for several widely used antibiotics remain only partially understood, complicating optimization and rational analog design [8]. Existing reviews typically address single drug classes or mechanisms in isolation, leaving a gap for integrative models that connect molecular action with bacterial physiology, resistance dynamics, and clinical outcomes.

In this review, we aim to fill this critical gap by systematically categorizing antibiotics based on their molecular targets, including cell wall synthesis, membrane integrity, nucleic acid replication, protein synthesis, and folate metabolism, while also mapping the associated resistance mechanisms. Beyond mechanistic analysis, we integrate emerging therapeutic strategies, including structure–guided drug design, adjuvant therapies, nanoparticle–based delivery, artificial intelligence–driven discovery, and precision medicine approaches. Through this comprehensive and multidisciplinary perspective, we aim to provide a unifying model that can guide both scientific research and clinical practices in tackling the AMR crisis.

## 2. Inhibitors of Cell Wall Biosynthesis

**Figure 1:**
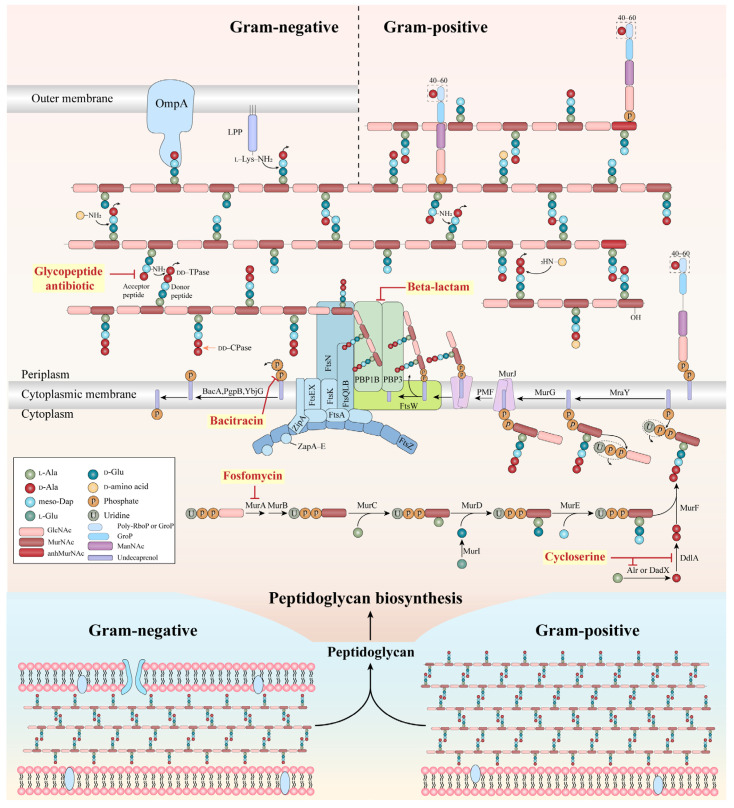
A comprehensive review of antibiotic targets involved in the process of peptidoglycan synthesis. The key enzymatic reactions involved in the synthesis and hydrolysis of peptidoglycan within the cell walls of both Gram–positive and Gram–negative bacteria are discussed, with a focus on the pentapeptide from *E. coli* as an illustrative example. Mur: muramyl ligase; Alr: alanine racemase; DadX: D–alanine racemase; Ddl: D–alanine ligase; Mra: phospho–N–acetylmuramoyl–pentapeptide–transferase; PMF: proton motive force; Fts: Filamenting temperature–sensitive mutan; PBP: penicillin–binding protein; Zip A: A type of inner–membrane protein; Zap A–E: FtsZ ring–associated proteins; YbjG: undecaprenyl–diphosphatase YbjG; PgpB: phosphatidylglycerophosphatase B; BacA: undecaprenyl–diphosphatase; DD–CPase: DD–carboxypeptidases; DD–Tpase: DD–transpeptidases; OmpA: outer membrane protein A. The synthesis of peptidoglycan is based on reference [11].

The bacterial cell wall is an essential structure that preserves cellular integrity, shape, and resistance to osmotic stress. Its main component, peptidoglycan (PG), consists of alternating N–acetylglucosamine (NAG) and N–acetylmuramic acid (NAM) units linked by β–(1,4) glycosidic bonds, with species–specific peptide stems cross linked to form a rigid three–dimensional matrix [9,10]. PG biosynthesis is a multistep pathway spanning cytoplasmic, membrane–associated, and periplasmic phases, involving more than 20 enzymes [11]. This complexity provides numerous antibacterial targets, making cell wall assembly one of the most exploited processes in antibiotic development. Several major antibiotic classes, including fosfomycin, D–cycloserine, β–lactams, glycopeptides, and bacitracin interfere with distinct stages of PG synthesis (Figure 1 and Table S1).

Inhibition of cytoplasmic precursor synthesis

### 2.1. Fosfomycin

Fosfomycin, a phosphonic acid derivative discovered in 1969, has been used clinically for over 40 years, mainly for uncomplicated urinary tract infections [12,13]. It exerts bactericidal activity by irreversibly inhibiting MurA, the enzyme catalyzing the first committed step of PG biosynthesis, through covalent binding as a structural analog of phosphoenolpyruvate [14,15]. By blocking PG precursor synthesis, fosfomycin disrupts cell wall integrity and leads to bacterial death [16]. Fosfomycin shows broad activity against Gram–negative pathogens, especially Enterobacteriaceae, and can penetrate biofilms, enhancing its utility in monotherapy and combination regimens against multidrug–resistant infections [17]. However, resistance develops through enzymatic inactivation (FosA, FosB), impaired uptake via GlpT/UhpT transporters, or MurA mutations [18]. To overcome this, current strategies include designing analogs less prone to enzymatic degradation, transporter–independent delivery systems, and rational combination therapies to restore efficacy.

### 2.2. D–Cycloserine

D–Cycloserine, discovered in 1955 from *Streptomyces* species, is a cell wall synthesis inhibitor with broad–spectrum activity [19]. The clinically relevant D–isomer blocks two essential cytoplasmic enzymes in PG biosynthesis: alanine racemase (Alr), which converts L–alanine to D–alanine, and D–alanine–D–alanine ligase (Ddl), which forms the D–Ala–D–Ala dipeptide required for PG crosslinking [20]. Simultaneously inhibiting both steps, it disrupts PG precursor formation and compromises cell wall integrity. Despite its antibacterial spectrum, cycloserine is mainly reserved for multidrug–resistant *Mycobacterium tuberculosis* due to dose–dependent neurotoxicity, which arises from off–target inhibition of mammalian enzymes involved in GABA metabolism [21]. This narrow therapeutic window limits its broader clinical use. Recent studies suggest stereoisomer–dependent pharmacology, with L–cycloserine showing higher potency and distinct kinetics compared to the D–isomer [22,23]. These findings highlight new opportunities for developing stereospecific derivatives, dual–target inhibitors, or formulations designed to reduce central nervous system exposure. Structure–guided optimization and rational combinations may help expand cycloserine’s therapeutic potential while mitigating neurotoxicity.

Inhibition of transglycosylation and transpeptidation

### 2.3. β–Lactam Antibiotics

β–Lactam antibiotics, defined by their four–membered β–lactam ring, represent a cornerstone of cell wall synthesis inhibitors and include penicillins, cephalosporins, cephamycins, monobactams, and carbapenems [24]. Their antibacterial activity relies on mimicking the D–Ala–D–Ala motif of peptidoglycan precursors, enabling covalent binding to penicillin–binding proteins (PBPs) and irreversible inhibition of transpeptidation, thereby disrupting peptidoglycan crosslinking and causing bacterial lysis [25,26]. The effectiveness of β–lactams is increasingly undermined by β–lactamases, classified as serine β–lactamases (SBLs) and metallo–β–lactamases (MBLs), with thousands of variants now identified [27]. To counteract this, β–lactamase inhibitors (BLIs) have been developed, which, although not bactericidal themselves, restore activity when combined with β–lactams and are now standard in clinical practice [28,29]. Current strategies emphasize next–generation BLIs with broader activity, particularly against MBLs, and novel β–lactam derivatives less prone to enzymatic hydrolysis. These advances are essential to preserve the clinical value of this foundational antibiotic class.

### 2.4. Glycopeptide Antibiotic

Glycopeptides inhibit cell wall synthesis by binding to the D–Ala–D–Ala termini of lipid II, thereby blocking trans–glycosylation and transpeptidation [30,31]. First–generation agents such as vancomycin and teicoplanin remain key treatments for Gram–positive infections, while second–generation derivatives like dalbavancin and oritavancin incorporate lipophilic modifications to enhance potency and membrane interaction [32]. Their spectrum is limited against Gram–negative bacteria due to outer membrane impermeability, and resistance against Gram–positive bacteria arises via replacement of D–Ala–D–Ala with D–Ala–D–Lac or D–Ala–D–Ser, which lowers binding affinity [33]. To overcome these barriers, strategies include designing polycationic derivatives with Gram–negative activity, nanoparticle delivery systems to improve tissue penetration, and biosynthetic engineering to generate structurally diversified analogs [31,34]. Integrating chemical modification, targeted delivery, and synthetic biology may expand their spectrum, overcome resistance, and reinstate glycopeptides as versatile therapeutics against evolving pathogens.

Inhibition of lipid carrier recycling

### 2.5. Bacitracin

Bacitracin, a cyclic polypeptide antibiotic first isolated from *Bacillus subtilis* in the 1940s, shows potent activity against Gram–positive bacteria and is mainly composed of bacitracin A [35]. It exerts bactericidal effects by binding undecaprenyl pyrophosphate (C55–PP), blocking its dephosphorylation to undecaprenyl phosphate (C55–P), an essential lipid carrier for peptidoglycan precursor transport [36]. This inhibition disrupts lipid carrier recycling and halts cell wall assembly. Due to systemic nephrotoxicity, bacitracin use is restricted to topical formulations for superficial infections, and its activity is limited to Gram–positive bacteria, with poor efficacy against Gram–negative bacteria [37]. Resistance, though relatively uncommon, involves the overexpression of lipid phosphatases or efflux systems such as BcrABC. To overcome these limitations, recent studies have explored bacitracin–functionalized nanomaterials and structural optimization to expand its antibacterial spectrum and reduce toxicity [38,39]. Future strategies should focus on rational lipid–carrier inhibitors with improved pharmacokinetics, combination regimens enhancing membrane penetration, and deeper insights into resistance pathways.

Other lipid carrier recycling inhibitors

Beyond bacitracin, several compounds disrupt lipid carrier recycling, though most remain preclinical. Friulimicin B, a cyclic lipopeptide from *Actinoplanes friuliensis*, inhibits BacA (UppP), which dephosphorylates undecaprenyl C55–PP to its active monophosphate form C55–P, thereby blocking carrier regeneration; however, poor pharmacokinetics limit its development [40]. Synthetic BacA/UppP inhibitors are also under exploration to selectively target pathogenic bacteria [41]. Some lantibiotics and novel peptides, such as nisin and teixobactin, act primarily by binding lipid II or related precursors, but they also indirectly impair lipid carrier turnover, offering resistance–evading advantages through non–protein targets. Despite encouraging in vitro activity, challenges including membrane permeability, toxicity, and delivery restrict clinical translation. Nonetheless, targeting lipid carrier recycling remains a promising antibacterial strategy, particularly when combined with nanodelivery systems or synergistic regimens to overcome Gram–negative barriers.

Mycobacterial cell wall inhibitors

**Figure 2:**
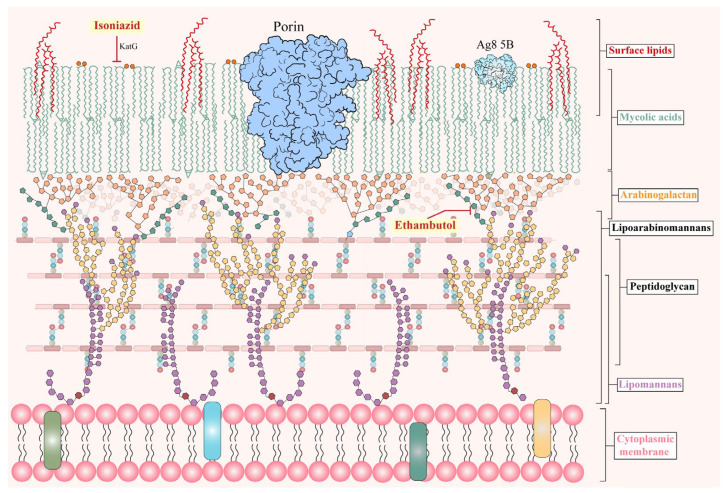
The cell wall and membrane of Mycobacterium bacteria and the antibiotic targets. Phosphoinositol mannosides are deep red for inositol and light purple for mannose. Arabinogalactan is dark green for galactan and light green for arabinan. Mycolic acids are indicated by light orange for α–cis/cis–cyclopropane and deep orange for keto–trans–cyclopropane. Phthiocerol dimycocerosate is red. Many classes of surface and cytoplasmic membrane lipids are not included. The cell wall structure is based on reference [42].

Unlike typical Gram–positive or Gram–negative bacteria, *M. tuberculosis* has a unique, lipid–rich cell envelope consisting of mycolic acids, arabinogalactan, and a dynamic cytoplasmic membrane, which confers intrinsic resistance to many antibiotics and demands specialized therapeutic strategies (Figure 2) [42]. Current frontline therapy exploits this vulnerability: isoniazid (INH) blocks mycolic acid synthesis, ethambutol (EMB) inhibits arabinogalactan assembly, rifampicin targets transcription, and pyrazinamide (PZA) disrupts energy metabolism and coenzyme A biosynthesis [43,44]. Combination regimens remain essential to ensure efficacy and suppress resistance. Given the global burden of tuberculosis and the complex mechanisms underlying drug action and resistance, a focused discussion is warranted [45]. In the following section, we outline the mechanisms of action and resistance pathways of INH and EMB, while rifampicin is discussed in the section on nucleic acid synthesis inhibitors.

### 2.6. Isoniazid

Isoniazid, synthesized in 1912, remains the cornerstone of tuberculosis therapy due to its potent and selective activity against M. tuberculosis. As a prodrug, INH requires activation by KatG to generate a nicotinyl–NAD adduct that inhibits InhA, the enoyl–ACP reductase of the FAS–II system, thereby blocking mycolic acid biosynthesis and compromising cell wall integrity [46,47]. Resistance is primarily driven by mutations in *katG* (notably S315T) as well as alterations in *inhA* and other genes, including *ahpC*, *kasA,* and *ndh*. Strategies to overcome resistance include direct InhA inhibitors that bypass KatG activation, KatG mimetics, and combination regimens that target multiple pathways. Elucidating alternative KatG–mediated activation products may further guide next–generation anti–tuberculosis drug development.

### 2.7. Ethambutol

Ethambutol, introduced in the 1960s, selectively inhibits mycobacterial arabinan biosynthesis by targeting arabinosyltransferases EmbA, EmbB, and EmbC, thereby impairing the mycolyl––arabinogalactan––peptidoglycan (mAGP) complex that maintains cell wall integrity [48]. Resistance is most often linked to *embB* mutations, particularly at codon 306, while *embA* and *embC* mutations occur less frequently [49]. Structural studies revealed that EMB competitively occupies the arabinose donor/acceptor site of Emb enzymes, disrupting arabinan elongation [50]. However, its clinical use is limited by its narrow spectrum and dose–dependent ocular toxicity. Future strategies include developing next–generation Emb inhibitors with improved specificity and reduced toxicity, as well as synergistic regimens that simultaneously impair multiple cell wall components.

## 3. Membrane–Disrupting Antibiotics

The bacterial cell membrane is essential for transport, signaling, and ion homeostasis, with structural differences between Gram–positive and Gram–negative species shaping therapeutic strategies [51]. A key distinction from mammalian membranes is the exposure of anionic lipids, which provides selective binding sites for cationic antimicrobial peptides (AMPs) [52]. AMPs are oligopeptides composed of 5–100 amino acid residues, and their secondary structures mainly adopt α–helical or β–sheet conformations [53,54].

**Figure 3:**
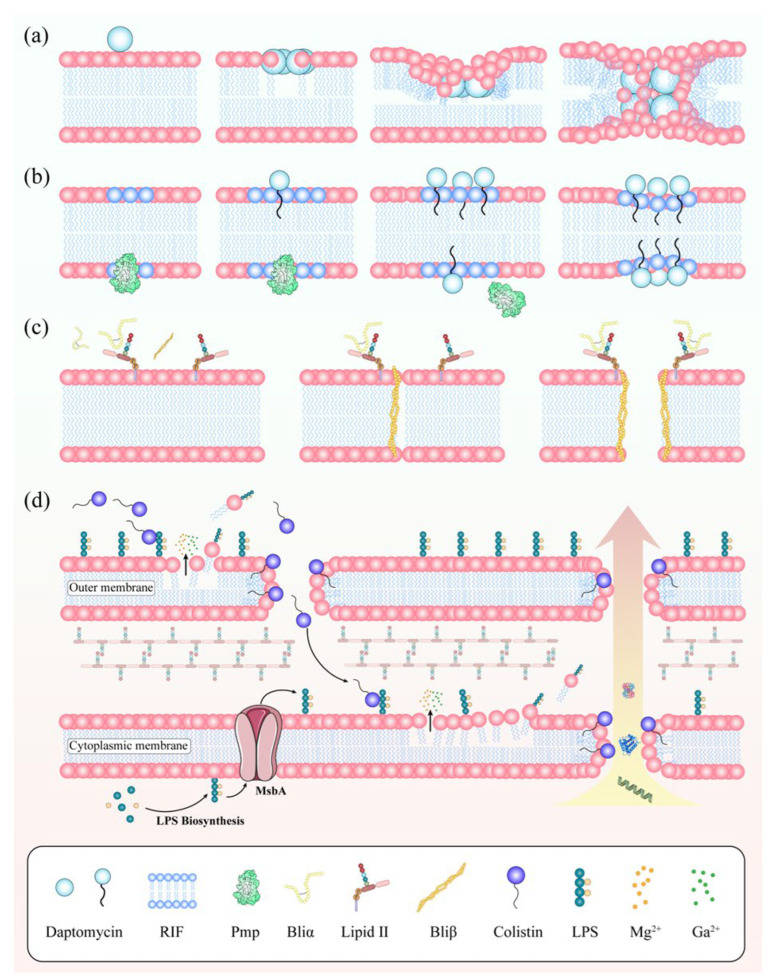
Mechanism of antibiotics targeting the cell membrane: (**a**) A model depicting the process of daptomycin insertion into and formation of pores in membranes. (**b**) A model illustrating the interaction between daptomycin and membranes, leading to protein delocalization. (**c**) Proposed dual–action mode of lichenicidin. Bliα recognizes and binds to lipid II, subsequently recruiting Bliβ to form a stable pore. (**d**) Proposed mechanism of action of PmE. PmE targets LPS in the periplasm by disrupting the outer membrane, resulting in leakage of cellular contents and bacterial death.

Although over 3000 AMPs have been identified, only a few peptide–based antibiotics have reached clinical use, such as daptomycin and polymyxin, largely due to limitations in stability, toxicity, and pharmacokinetic properties [55,56]. Nevertheless, their membrane selectivity and multifaceted mechanisms make them a continuing focus for antibiotic innovation. The following sections highlight representative AMPs and their modes of action (Figure 3 and Table S2).

### 3.1. Daptomycin

Daptomycin, a cyclic lipopeptide with a fatty acid tail, is the only membrane–targeting antibiotic approved for systemic use against Gram–positive infections [57]. Its bactericidal activity is calcium–dependent. Ca^2+^ binding induces conformational changes that increase amphipathicity and promote insertion into negatively charged, phosphatidylglycerol–rich membranes [58]. Although initially thought to inhibit peptidoglycan or lipoteichoic acid synthesis, daptomycin is now recognized to act directly on membranes [59]. In vitro studies show that Ca^2+^–dependent oligomers form in bacterial membranes, creating cation–selective pores that depolarize the membrane (Figure 3a). However, pore formation alone does not fully account for its specificity or potency. Recent work highlights a broader mechanism involving the disruption of membrane lipid organization. Daptomycin targets regions of increased fluidity (RIF), rigidifying these microdomains and displacing peripheral membrane proteins such as MurG (lipid II synthase) and PlsX (phospholipid synthase). This impairs cell wall synthesis, membrane function, and division (Figure 3b) [57]. Thus, daptomycin likely exerts its activity through the combined effects of pore formation and domain rigidification. Despite its clinical success, resistance remains a concern, and the precise contributions of pore formation, lipid domain targeting, and amino acid composition to activity are incompletely understood. Future efforts should integrate lipidomics, structural biology, and imaging approaches to refine mechanistic models and guide the rational design of next–generation lipopeptides with enhanced potency, expanded spectrum, and reduced resistance potential.

### 3.2. Lantibiotics

Lantibiotics are ribosomally synthesized, post–translationally modified peptides produced by Gram–positive bacteria, characterized by unusual amino acids (lanthionine, MeLan, Dha, and Dhb) that form thioether bridges conferring stability and bioactivity [60]. They are generally <5 kDa and classified as Type A (elongated, pore–forming, e.g., nisin) or Type B (globular, enzyme inhibitors) [61]. Their primary target is lipid II, a key peptidoglycan precursor [62]. Type A lantibiotics, such as nisin, form lipid II–peptide complexes that generate pores and disrupt membranes, while others sequester lipid II to block cell wall synthesis without pore formation [63]. Dual–peptide lantibiotics (e.g., lichenicidin) employ cooperative mechanisms where one subunit binds lipid II, and the other mediates pore formation (Figure 3c) [64]. Resistance, though less common than with conventional antibiotics, arises through lipid II modifications, membrane charge remodeling (MprF–mediated lysinylation), efflux pumps (NisFEG, SpaFEG), or proteolytic degradation. To overcome these challenges, strategies include structure–guided engineering to enhance lipid II affinity and resistance evasion, hybrid molecules combining lantibiotic scaffolds with other pharmacophores, and co–administration with efflux or protease inhibitors [65]. Advances in nanoparticle delivery and synthetic biology platforms further enable improved stability, targeted delivery, and scalable production of novel analogs. Collectively, these approaches highlight lantibiotics as promising membrane–active agents with unique mechanisms and opportunities for clinical translation.

### 3.3. Polymyxin

Polymyxins, including polymyxin B (PmB) and polymyxin E (colistin), are cyclic lipopeptides reserved as last–line agents against multidrug–resistant (MDR) Gram–negative pathogens due to their nephrotoxicity and neurotoxicity. Structurally, they are amphipathic decapeptides containing five cationic diaminobutyric acid residues and an N–terminal fatty acyl chain, enabling strong interaction with lipopolysaccharide (LPS). By displacing divalent cations (Mg^2+^, Ca^2+^) that stabilize LPS, polymyxins insert into lipid A, destabilizing membranes and causing bacterial death [66,67]. Beyond pore formation, polymyxins disrupt membrane architecture by altering curvature, rigidifying microdomains, and impairing protein function [68,69]. In vivo, colistin (PmE) can traverse the outer membrane to disrupt inner–membrane LPS and trigger cytoplasmic rupture [70], while simulations suggest polymyxin B may aggregate on membrane surfaces, modulate rigidity, and induce vesicle interactions without full insertion [71,72]. Resistance largely arises from lipid A modification with L–Ara4N or phosphoethanolamine, mediated by chromosomal two–component systems or plasmid–borne *mcr* genes, which reduce membrane negative charge and polymyxin binding. Additional mechanisms include efflux pumps and capsule overproduction (Figure 3d). To address these challenges, next–generation polymyxin derivatives (e.g., SPR741, QPX9003) show reduced toxicity with retained efficacy. Combination therapies with rifampicin, carbapenems, or LPS transport inhibitors can resensitize resistant strains, while advances in stereochemistry, structural biology, and molecular simulations are guiding the rational design of optimized analogs through noncanonical amino acids, lipid–tail diversification, and sequence tuning. Innovative delivery platforms, including nanoparticles and prodrugs, further enhance pharmacokinetics and safety. Polymyxins remain indispensable for MDR Gram–negative infections, but their sustained clinical utility will rely on integrating these molecular, therapeutic, and delivery strategies to overcome resistance and toxicity barriers.

### 3.4. Short Peptides and Other Compounds

Beyond natural cyclic peptides, synthetic short peptides (<5 amino acids) and AMP mimetics have been developed to overcome limitations of stability and toxicity [73]. Despite their minimal size, compounds such as Compound 1, Compound 2, and Ltx5 retain potent antibacterial activity, underscoring that efficacy depends more on the amphiphilic presentation of cationic and hydrophobic residues than sequence complexity [74,75]. These agents generally disrupt membrane integrity and homeostasis, with depolarization, rather than full lysis, often sufficient for bactericidal action. Optimization strategies include chemical modifications such as halogenation, which enhances potency and modulates membrane interactions while sometimes conferring additional anti–inflammatory or anticancer activity [76]. Non–peptidic mimetics, including ceragenins, reutericyclin, polyene macrolides, and xanthone derivatives, similarly destabilize membranes, while amphiphilic aminoglycoside derivatives expand antibacterial spectra through added membrane–permeabilizing effects [77,78,79,80]. A unifying feature is their interaction with divalent cations (Mg^2+^, Ca^2+^), perturbing proton motive force and membrane–associated physiology beyond simple disruption. Although resistance remains underexplored, potential mechanisms include altered lipid composition, charge, or fluidity. Future strategies should emphasize structure––activity–guided design, incorporation of noncanonical residues, stereochemical tuning, and targeted delivery systems to enhance selectivity, reduce toxicity, and exploit membrane homeostasis as a therapeutic vulnerability.

## 4. Inhibitors of Nucleic Acid

**Figure 4:**
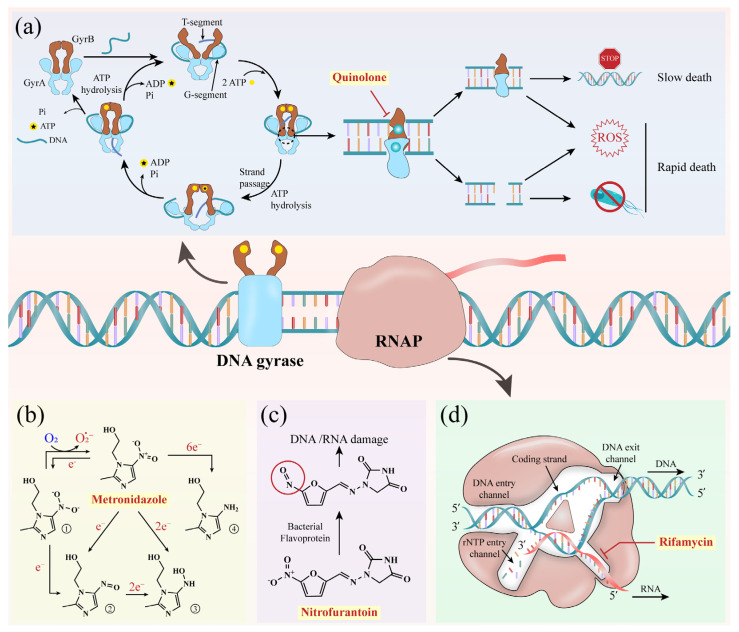
The antibiotics disrupt bacterial nucleic acid synthesis and replication: (**a**) The catalytic cycle of DNA supercoiling induced by DNA gyrase and the mechanism of action of quinolones. The specific stages of the catalytic cycle reaction on the left are unclear, with the dashed black circles and the right inset illustrating the lethal model of quinolones. Quinolones stabilize the drug––enzyme––DNA complex between the DNA double strands, affecting replication and transcription, ultimately leading to slow bacterial death if the complex is not cleared. If the broken double strands are not repaired after removing the complex, it results in rapid bacterial death. The presence of quinolones also causes ROS accumulation, leading to rapid bacterial death. (**b**) Reduction and toxicity of metronidazole in bacteria. Depending on the number of electrons accepted by metronidazole, it can form a nitroimidazole radical anion ①, a nitrosoimidazole ②, and a hydroxylaminimidazole ③. This process can proceed in the sequence ①→③ or be achieved in a single catalytic step. It is hypothesized that certain enzymes can transfer 6e– to metronidazole to form the non–reactive aminoimidazole ④, leading to metronidazole resistance. In the presence of oxygen, ① can be oxidized back to the metronidazole precursor. Active intermediates ①–③ damage bacterial DNA. Molecular oxygen is shown in blue; the superoxide radical and electrons are shown in red. (**c**) The bacterial flavoproteins convert nitrofurans to the corresponding nitro compounds, causing damage to bacterial DNA/RNA. The red circle highlights the reduced nitroso intermediate produced by bacterial flavoproteins. (**d**) Rifamycins inhibit RNA synthesis by directly blocking the RNA extension pathway by binding to RNA polymerase (RNAP).

Bacterial nucleic acid synthesis depends on tightly regulated DNA topology and RNA polymerase activity. Type II topoisomerases, namely DNA gyrase and topoisomerase IV, are essential. Gyrase uniquely introduces negative supercoils while relaxing positive ones, whereas topoisomerase IV primarily resolves catenanes and aids in supercoil relaxation [81]. RNA polymerase, directly responsible for transcription, represents another critical target. Antibiotics acting on these enzymes effectively block replication or transcription. In what follows, we outline major nucleic acid synthesis inhibitors, including agents targeting DNA gyrase/topoisomerase IV and those directly inhibiting RNA synthesis, such as rifamycins (Figure 4 and Table S3).

### 4.1. Quinolones

Quinolones, originating with nalidixic acid in the 1960s, are broad–spectrum agents now dominated by fluoroquinolones (FQs), which feature a quinoline core and fluorine substitutions enhancing potency [82,83]. The WHO designates FQs as critically important due to their clinical success. Their primary targets are DNA gyrase and topoisomerase IV, where quinolones trap a drug––enzyme––DNA cleavage complex, stalling replication and transcription [84]. At near–MIC levels, inhibition is mainly bacteriostatic, while higher concentrations drive irreversible DNA breaks and rapid cell death [85]. Additional mechanisms include degradation of topoisomerase subunits and reactive oxygen species (ROS) accumulation, which exacerbate DNA and membrane damage, though the contribution of ROS remains debated (Figure 4a) [86,87]. Resistance has become widespread, driven by mutations in quinolone resistance–determining regions (QRDRs) of GyrA/ParC, efflux pump overexpression, and plasmid–mediated Qnr proteins that shield topoisomerases [88,89]. To counter this, strategies focus on next–generation quinolones with enhanced binding, adjuvants that block efflux or boost ROS, and mechanistic studies to guide rational design. Despite rising resistance, quinolones remain indispensable, provided their use is safeguarded by surveillance, innovation, and combination therapies.

### 4.2. Metronidazole

Metronidazole, a nitroimidazole prodrug, is a first–line agent against anaerobic bacteria and protozoa such as *Clostridioides difficile* and *Giardia lamblia* [90]. Its selective toxicity arises from intracellular reduction under anaerobic conditions, mediated by enzymes like pyruvate: ferredoxin oxidoreductase and thioredoxin reductase, producing reactive intermediates that damage DNA through strand breaks and synthesis inhibition (Figure 4b) [91,92]. Despite decades of clinical use, metronidazole remains the most important nitroimidazole; newer analogs such as tinidazole and ornidazole mainly improve pharmacokinetics without altering the mechanism. Resistance, though still uncommon, is increasing in *H. pylori* and *C. difficile*, driven by impaired drug activation, enhanced DNA repair, and antioxidant defenses [91]. Major limitations include its strict dependence on anaerobic metabolism and incomplete knowledge of activation pathways, which hinder rational derivative design. Future strategies focus on identifying key reductive enzymes, developing analogs with oxygen–independent activation, and combining metronidazole with redox modulators or DNA repair inhibitors to broaden its spectrum and counter resistance.

### 4.3. Nitrofurantoin

Nitrofurantoin, the only nitrofuran approved for human use, is widely employed for treating and preventing lower urinary tract infections [93,94]. As a prodrug, it is reduced by bacterial nitroreductases (NfsA, NfsB) to reactive intermediates that damage DNA and RNA, inhibit protein synthesis, disrupt the TCA cycle, and impair ATP production, cumulatively overwhelming bacterial repair systems (Figure 4c) [95,96]. Its clinical efficacy remains high, especially against E. coli, because resistance requires loss–of–function mutations in nitroreductases, changes that also reduce bacterial fitness [97]. Nonetheless, resistance via *nfsA* and *nfsB* mutations has been reported. Limitations include poor tissue penetration, urinary tract confinement, and reduced efficacy in patients with renal impairment. Future strategies aim to develop analogs with broader distribution and activation independent of nitroreductases or to combine nitrofurantoin with agents enhancing oxidative or metabolic stress. A deeper mechanistic understanding will be essential to extend its utility and inspire next–generation nitrofuran therapies.

### 4.4. Rifamycin

Rifamycins, polyketide–derived ansamycins with a naphthalene core and macrocyclic bridge, are potent bactericidal agents mainly against Gram–positive bacteria and mycobacteria. Clinically, rifampicin and rifapentine are essential for tuberculosis therapy, while rifabutin and rifaximin are used for *Mycobacterium avium* complex infections and traveler’s diarrhea [98,99]. Their activity stems from high–affinity binding to the β–subunit of bacterial RNAP, where they block transcription initiation by preventing the formation of the first phosphodiester bonds, leaving elongation largely unaffected (Figure 4d) [100]. Selectivity over eukaryotic RNAP minimizes host toxicity [101]. Resistance arises rapidly via *rpoB* mutations in the RNAP β–subunit, which alter rifamycin–binding residues while preserving enzyme function [102]. This single–target vulnerability is a major limitation, particularly in tuberculosis therapy, where monotherapy quickly selects resistant strains. Efforts to overcome resistance focus on structural innovation. Kanglemycins, with ansa–bridge sugar modifications, retain or restore activity against rifampicin–resistant mutants by engaging alternative RNAP residues. Combination therapies with drugs targeting non–overlapping RNAP sites or resistance pathways are also under exploration. Sustaining rifamycins’ clinical utility will require structure–guided analog development and integrated therapeutic strategies to counter mounting resistance.

## 5. Inhibitors of Protein Synthesis

**Figure 5:**
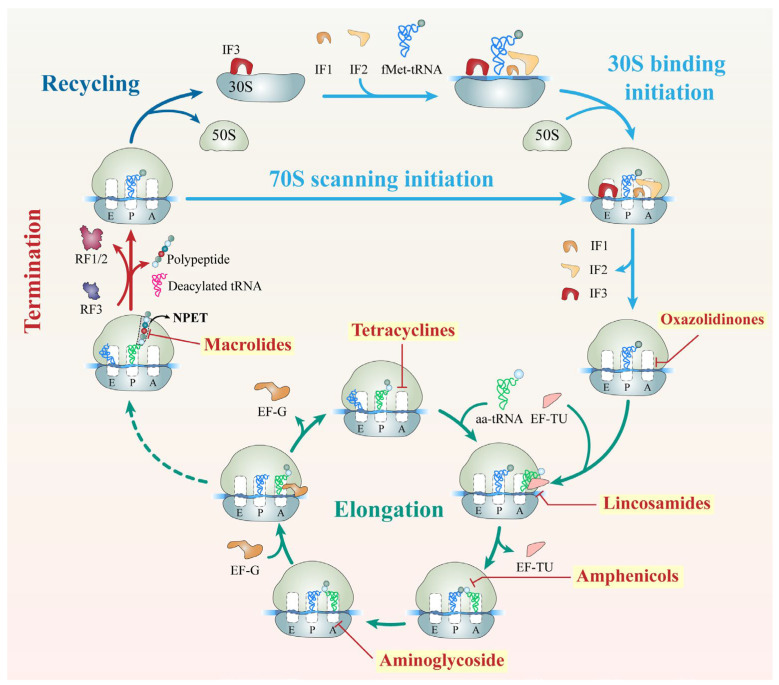
An overview of antibiotic target sites in the bacterial translation process. For simplicity, only the essential details of the translation process are presented, focusing on antibiotic binding to ribosomes to inhibit bacterial translation. Different stages of the translation cycle are distinguished by different colors. The dashed arrow signifies the functional transition from the completion of the Elongation cycle to the commencement of the Termination phase, which is typically triggered when the ribosome encounters a stop codon in the A-site. IF: initiation factor; tRNA: transfer RNA; fMet–tRNA: tRNA modified with N–formylmethionine; aa–tRNA: aminoacyl–tRNA; EF: elongation factor; RF: release factor; NEPT: nascent peptide exit tunnel.

Protein synthesis, an energy–intensive and highly regulated process, is mediated by the bacterial 70S ribosome, composed of the 30S and 50S subunits. Translation proceeds through initiation, elongation, termination, and recycling, coordinated by the ribosome’s functional centers: the decoding center (DC) in the 30S subunit ensures codon–anticodon accuracy, while the peptidyl transferase center (PTC) in the 50S subunit catalyzes peptide bond formation [103,104]. Given its essential role, the ribosome is one of the most prolific antibiotic targets. Many clinically important agents inhibit translation by binding to specific ribosomal sites, disrupting distinct stages of protein synthesis. These inhibitors are broadly divided into 50S–targeting antibiotics (e.g., oxazolidinones, phenicols, macrolides, and lin–cosamides) and 30S–targeting antibiotics (e.g., tetracyclines, aminoglycosides). The following sections summarize major ribosome–targeting antibiotics, their mechanisms of action, structural basis of binding, resistance mechanisms, and strategies to overcome resistance (Figure 5 and Table S4).

### 5.1. Oxazolidinones

Oxazolidinones are among the few truly novel synthetic antibiotic classes introduced in recent decades, with no natural homologs. Linezolid, approved in 2000, provided a crucial option against multidrug–resistant Gram–positive pathogens such as MRSA and vancomycin–resistant *Enterococcus*, while the second–generation tedizolid (2014) offered improved potency, safety, and once–daily dosing. Their antibacterial activity arises from binding the PTC of the 50S ribosomal subunit, where they block aminoacyl–tRNA accommodation and peptide bond formation [105]. Recent studies indicate that this inhibition is sequence–dependent. Linezolid preferentially stalls ribosomes at specific alanine–serine/threonine motifs but is less effective in the presence of glycine, suggesting oxazolidinones act not as universal PTC blockers but through context–specific interactions with the nascent peptide [106,107]. Resistance is increasingly reported, mainly via mutations in 23S rRNA or acquisition of the *cfr* methyltransferase, which modifies the ribosomal binding site. To overcome this, next–generation derivatives are being developed through structure–guided design to enhance ribosomal affinity and evade resistance, ensuring oxazolidinones remain vital in treating resistant Gram–positive infections.

### 5.2. Amphenicols

Amphenicols, typified by chloramphenicol from *Streptomyces venezuelae*, inhibit protein synthesis by targeting the PTC of the 50S ribosomal subunit. Traditionally thought to block A–site tRNA accommodation, more recent evidence shows their inhibition depends on nascent peptide context. Chloramphenicol binds weakly to vacant ribosomes but stabilizes once a nascent chain of 3–6 residues forms new interfaces in the nascent peptide exit tunnel (NPET). Specific penultimate residues, alanine, serine, or threonine, promote ribosome stalling, highlighting a tripartite interaction among the drug, ribosome, and peptide. Thus, chloramphenicol functions as a context–specific rather than purely competitive PTC inhibitor [104]. This mechanism closely parallels that of oxazolidinones, despite structural differences, underscoring a broader principle of nascent chain–modulated translation inhibition. Resistance arises mainly through chloramphenicol acetyltransferases, efflux pumps, or ribosomal mutations, while toxicity has further constrained clinical use. Nevertheless, the unique ribosomal interactions of amphenicols continue to inform the design of new PTC inhibitors with enhanced specificity and reduced resistance.

### 5.3. Macrolides

Macrolides, typified by erythromycin, are natural product antibiotics featuring a 12–16 carbon macrocyclic lactone ring glycosylated with desosamine and cladinose sugars. Clinically important members include 14– and 15–membered macrolides used in humans, while 16–membered forms are mainly veterinary. Structural modifications of erythromycin led to ketolides (e.g., telithromycin), which improve ribosomal binding and activity against resistant strains [108]. Macrolides act by binding the ribosomal NPET, traditionally viewed as steric “plugs” halting elongation. However, structural and ribosome–profiling studies reveal a more nuanced mechanism: macrolides induce sequence–specific translational stalling at defined motifs, termed macrolide arrest motifs (MAMs), with distinct selectivity between erythromycin and ketolides [109]. This positions macrolides as context–dependent modulators of protein synthesis, rather than global inhibitors. Resistance arises mainly from methylation of 23S rRNA, efflux pumps, or enzymatic inactivation. Current strategies focus on next–generation derivatives with optimized NPET interactions, novel macrolide scaffolds, and combination regimens to overcome resistance. A deeper understanding of MAM–guided stalling may enable the precision design of macrolides that selectively block pathogenic protein expression.

### 5.4. Lincosamides

Lincosamides are composed of an amino acid moiety (L–proline or its 4′–alkyl–substituted derivatives) linked to a methylthiolincosamide core [110]. Lincomycin, first isolated from *Streptomyces lincolnensis*, was later improved by structural modification to yield clindamycin, which remains the clinically dominant derivative [111]. Mechanistically, lincosamides bind the PTC of the 50S ribosomal subunit. By forming a hydrogen–bonding network with 23S rRNA in the A site, they block stable aminoacyl–tRNA accommodation and thereby inhibit peptide bond formation [112]. Their antibacterial activity critically depends on structural features: the thiooctose moiety with its S–alkyl substituent is indispensable, while the N–methyl–PPL/Pro linkage and variations in alkyl side chain length or salicylate ester substitutions modulate potency, with longer side chains often enhancing activity [113,114]. Resistance is mainly mediated by 23S rRNA methylation (*erm* genes), efflux pumps, or enzymatic inactivation. Future development focuses on optimizing ribosomal binding and structural scaffolds to broaden their spectrum and overcome resistance, guided by deeper insights into PTC interactions and nascent peptide context.

### 5.5. Tetracyclines

Tetracyclines are broad–spectrum antibiotics defined by a linear tetracyclic core with variable functional groups. The first members, chlortetracycline and oxytetracycline, paved the way for improved derivatives such as doxycycline and minocycline, which remain in use today [115,116]. They act as bacteriostatic agents by binding the 30S ribosomal subunit’s decoding center, where their C and D rings sterically block aminoacyl–tRNA accommodation at the A site, inhibiting codon recognition and peptide elongation [117]. Cellular uptake depends on Mg^2+^ interactions: in Gram–negative bacteria, tetracyclines cross porins as Mg^2+^ complexes and then diffuse across the inner membrane, while Gram–positive uptake involves both passive and active transport; intracellularly, they re–chelate Mg^2+^ to bind the ribosome [118]. Resistance, mainly via efflux pumps and ribosomal protection proteins, has limited the early tetracyclines. To address this, third–generation glycylcyclines were developed, with tigecycline showing >100–fold stronger ribosomal binding than first–generation agents and broad activity against resistant strains [118,119]. Current efforts emphasize structural optimization to counter resistance and improve pharmacokinetics. A deeper understanding of ribosome interactions and resistance pathways will be crucial for guiding next–generation tetracycline development.

### 5.6. Aminoglycosides

Aminoglycosides (AGAs) are potent bactericidal antibiotics originally discovered from soil microorganisms, with streptomycin from *Streptomyces* as the first representative [120]. They consist of amino sugars linked to an aminocyclitol core, mainly 2–deoxystreptamine (2–DOS), and are structurally classified into four groups: DOS–free (e.g., streptomycin), monosubstituted DOS (e.g., apramycin), 4,5–disubstituted DOS (e.g., neomycin), and 4,6–disubstituted DOS (e.g., gentamicin) [121]. Their antibacterial activity depends on uptake into bacterial cells and binding to the 30S ribosomal subunit, where they interact with the A–site of 16S rRNA to induce codon misreading and the incorporation of incorrect amino acids. Structural studies show that aminoglycosides such as paromomycin fit into the A–site RNA groove, with N1 and N3 amino groups on the 2–DOS ring stabilizing binding and altering decoding fidelity. This leads to misincorporation, frameshifting, and ORF corruption, ultimately producing dysfunctional proteins. Some AGAs also bind eukaryotic decoding centers, contributing to off–target toxicity. Recent ribosome profiling reveals that AGAs like kanamycin A and sisomicin promote clusters of misreading across elongation cycles, with mistranslation probabilities up to 40%, explaining how even sub–inhibitory levels disrupt translation and enhance antibiotic efficacy [122]. Resistance arises mainly from aminoglycoside–modifying enzymes, reduced uptake, and ribosomal mutations. Current development focuses on next–generation AGAs with stronger ribosomal binding, lower toxicity, and activity against resistant pathogens.

## 6. Metabolic Pathway Inhibitors: Targeting Folate Biosynthesis

Folate metabolism provides essential one–carbon units for nucleotide and amino acid synthesis, and unlike eukaryotes, bacteria synthesize folate de novo, making this pathway an attractive antibiotic target [123,124]. The pathway culminates in tetrahydrofolate (THF), a cofactor critical for DNA and RNA precursor production [125,126]. Two enzymes represent the primary antibacterial targets: dihydropteroate synthase (DHPS), inhibited by sulfonamides, and dihydrofolate reductase (DHFR), inhibited by diaminopyrimidines [127,128]. These inhibitors exploit a fundamental prokaryote––eukaryote metabolic distinction, achieving selective toxicity by indirectly suppressing nucleic acid synthesis and cell proliferation.

**Figure 6:**
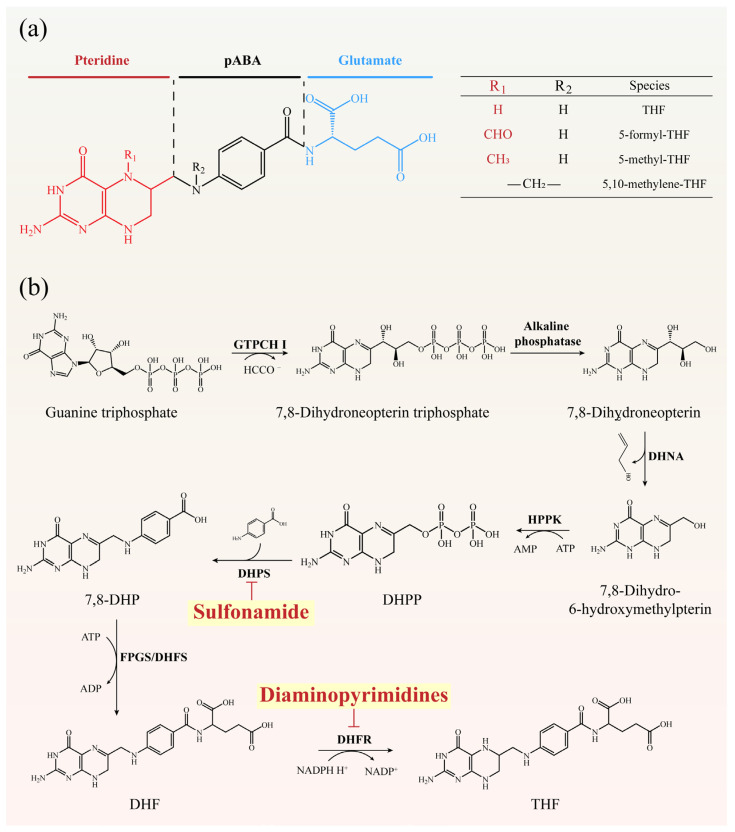
Folate derivatives and biosynthesis: (**a**) Chemical composition of folic acid. (**b**) Overview of the folate pathway in microorganisms. GTPCH I: GTP cyclohydrolase I; DHNA: dihydroneopterin aldolase; HPPK: 7,8–dihydro–6–hydroxymethylpterin–pyrophosphokinase; DHPS: dihydropteroate synthase; FPGS: folate polyglutamate synthase; DHFS: dihydrofolate synthase; DHFR: dihydrofolate reductase.

This section highlights the mechanisms, clinical relevance, and resistance challenges of folate biosynthesis inhibitors, illustrating how metabolic targeting complements ribosome– and nucleic acid–directed antibiotics (Figure 6).

### 6.1. Sulfonamide

Sulfonamides are synthetic antimicrobials defined by a sulfonamide group para–substituted with an amino group, a structural feature essential for activity. By mimicking p–aminobenzoic acid (pABA), they competitively inhibit DHPS, preventing the condensation of pABA with dihydropterin pyrophosphate and blocking THF synthesis. This interruption of folate metabolism suppresses nucleotide production, leading to bacteriostasis through inhibition of DNA replication and cell division [129]. Clinical efficacy has declined due to widespread resistance, mainly from mutations in *folP* (DHPS gene), acquisition of resistant DHPS variants via horizontal transfer, or increased pABA production that reduces drug competition. Current approaches to restoring activity include designing novel DHPS inhibitors with higher affinity for resistant enzymes and combination regimens with DHFR inhibitors (e.g., trimethoprim) that block sequential steps of folate metabolism. Additional strategies under investigation involve dual–target inhibitors acting on multiple enzymes within the pathway or adjuvants that restore sulfonamide sensitivity.

### 6.2. Diaminopyrimidines

Diaminopyrimidines target the second key step of folate metabolism by inhibiting DHFR, which catalyzes the NADPH–dependent reduction of DHF to THF, thereby blocking thymidine and purine biosynthesis essential for DNA replication. The best–known agent, trimethoprim, is widely used alone or in combination with sulfonamides, where the sequential blockade of DHPS and DHFR produces strong synergy. However, resistance has become common, driven by mutations in *folA* (DHFR gene) or acquisition of resistant DHFR variants via horizontal gene transfer, both of which reduce drug binding while preserving enzyme activity [130]. Counterstrategies include rational design of next–generation DHFR inhibitors guided by structural analyses of resistant variants, development of broad–spectrum inhibitors with enhanced binding, and renewed emphasis on combination therapy with DHPS inhibitors. Emerging approaches also explore dual–target compounds and adjuvants that can bypass or neutralize resistance mechanisms, potentially rejuvenating this drug class.

## 7. Emerging Strategies to Combat Antimicrobial Resistance

**Figure 7:**
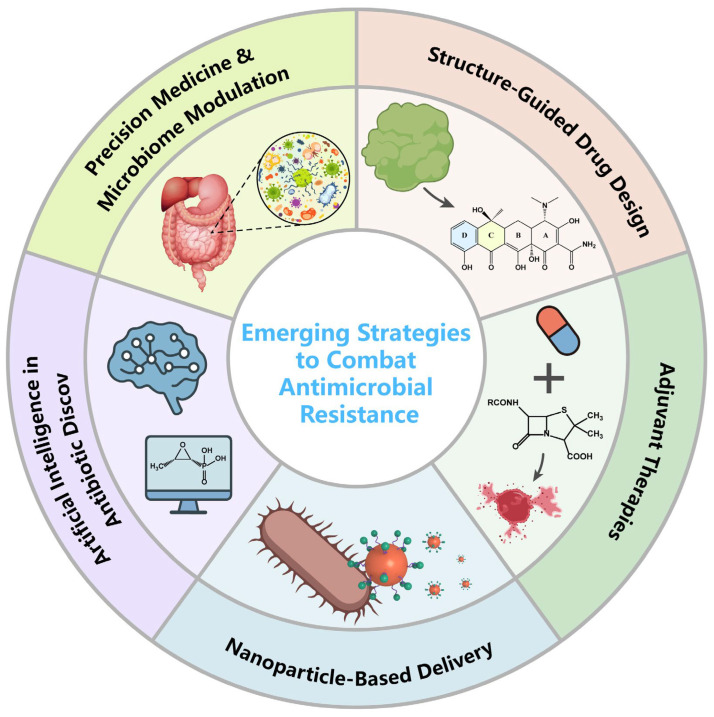
Technological innovations and therapeutic modalities in the development of anti-resistance tools.

The relentless rise of antimicrobial resistance, coupled with the slow pace of new antibiotic discovery, demands innovative strategies that transcend traditional approaches. While earlier sections highlighted drug–specific improvements such as structural refinements, delivery innovations, and combination regimens, this section synthesizes broader, interdisciplinary strategies that are reshaping the future of antimicrobial therapy. Current efforts converge across five major domains: structure–guided drug design, adjuvant therapies, nanotechnology–based delivery, artificial intelligence, and precision medicine with microbiome modulation (Figure 7).

### 7.1. Structure–Guided Drug Design

Building on the mechanistic insights discussed in earlier sections, structure–guided drug design leverages crystallography, cryo–EM, and molecular modeling to optimize antibiotic–target interactions. Recent examples include inhibitors of Nocardia NsdUTPase and S. aureus SaCntL, which demonstrate improved affinity and potency compared with marketed drugs. Looking ahead, the integration of structural biology with AI–driven modeling and multi–omics is expected to accelerate rational antibiotic development and resistance evasion [56].

### 7.2. Adjuvant Therapies

Adjuvants extend the lifespan of existing antibiotics by restoring activity against resistant pathogens or enhancing drug potency [131]. Classic β–lactamase inhibitors exemplify this approach, while novel candidates such as indole derivatives, flavonoids, and host defense peptides show potential to increase antibiotic efficacy by up to 64–fold. Efflux pump inhibitors like verapamil have also demonstrated clinical benefit in combination regimens [132]. These strategies illustrate how “drug repurposing” and precision combinations can maximize therapeutic outcomes while mitigating resistance.

### 7.3. Nanoparticle–Based Delivery

Nanotechnology offers a versatile platform to improve antibiotic pharmacokinetics, tissue distribution, and intracellular penetration, particularly against biofilm–associated or intracellular infections [133,134]. For example, mesoporous silica nanoparticles have enabled targeted cefoperazone delivery against MRSA [135], while liposomal ciprofloxacin formulations have improved therapeutic efficacy in clinical trials [136,137]. Such delivery systems not only enhance drug stability and reduce toxicity but also represent a promising route for revitalizing antibiotics limited by poor solubility or unfavorable distribution.

### 7.4. Artificial Intelligence in Antibiotic Discovery

Artificial intelligence (AI) has revolutionized antibiotic discovery by enabling high–throughput virtual screening, structural optimization, and target validation [138]. Deep learning has already yielded novel scaffolds such as halicin, and large–scale in silico screening has identified multiple candidates from vast molecular libraries [139]. Coupled with tools like AlphaFold, AI has provided structural insights into resistance–associated proteins such as efflux pumps, guiding rational inhibitor design. As experimental validation pipelines become increasingly integrated with AI–driven workflows, the pace of novel antibiotic discovery is expected to accelerate dramatically.

### 7.5. Precision Medicine and Microbiome Modulation

Beyond drug design, precision medicine seeks to tailor antimicrobial therapy based on pathogen genomics, host responses, and microbiome composition [140]. Recent studies highlight how antibiotic–induced dysbiosis, such as vancomycin–mediated gut microbiota disruption, undermines therapeutic outcomes. Emerging strategies combine genomic profiling with bacteriophage libraries or microbiome–based interventions to personalize therapy and preserve microbial homeostasis [141]. Such approaches aim not only to treat infections more effectively but also to reduce collateral damage to the host ecosystem.

## 8. Conclusions

The optimism of the antibiotic golden age that infectious diseases could soon be eradicated has been tempered by the relentless rise of antimicrobial resistance and the slowdown of pharmaceutical innovation. A recent WHO report underscores the urgency: since 2017, only 13 new antibiotics have been approved, and just two represent novel mechanisms. This stagnation highlights the continued reliance on traditional antibiotic classes as critical tools in combating bacterial infections.

In this review, we have systematically examined the mechanisms of action across major antibiotic classes, emphasizing how molecular targets, modes of action, and structural features inform strategies to counter resistance. While incremental improvements to existing drugs remain indispensable, the path forward will increasingly depend on integrating structural biology, multi–omics, and artificial intelligence to accelerate rational drug design.

Ultimately, a mechanism–driven research paradigm, grounded in knowledge of both established and emerging agents, will be central to developing precision therapies and overcoming resistance. Achieving this vision demands sustained scientific innovation, interdisciplinary collaboration, and robust global investment. Only then can we preserve the effectiveness of antibiotics and safeguard human health in the post–antibiotic era.

## Data Availability

No new data were created or analyzed in this study. Data sharing is not applicable to this article.

## Supplementary Materials

The following supporting information can be downloaded at: https://www.mdpi.com/article/10.3390/antibiotics15040351/s1. Table S1: Chemical structure and mechanism of action of antibiotics target cell wall; Table S2: Chemical structures and mechanisms of action of antibiotics targeting the cell membrane; Table S3: Chemical structures and mechanisms of action of antibiotics that inhibit nucleic acid synthesis; Table S4: Chemical structures and mechanisms of action of antibiotics that inhibit protein synthesis. Refs. [20,50,57,64,66,73,74,75,82,84,90,94,98,104,105,109,111,115,120,142,143,144,145,146,147,148,149] are cited in Supplementary Materials.

## Abbreviations

The following abbreviations are used in this manuscript:

AMR	Antimicrobial resistance
GLASS	Global Antimicrobial Resistance and Use Surveillance System
MRSA	Methicillin–resistant *Staphylococcus aureus*
PG	Peptidoglycan
NAG	N–acetylglucosamine
NAM	N–acetylmuramic acid
Alr	Alanine racemase
Ddl	D–alanine–D–alanine ligase
PBPs	Penicillin–binding proteins
SBLs	Serine β–lactamases
MBLs	Metallo–β–lactamases
BLIs	Β–lactamase inhibitors
C55–PP	Undecaprenyl pyrophosphate
C55–P	Undecaprenyl phosphate
UppP	Undecaprenyl pyrophosphate phosphatase
INH	Isoniazid
EMB	Ethambutol
PZA	Pyrazinamide
mAGP	Mycolyl–arabinogalactan–peptidoglycan
AMPs	Antimicrobial peptides
RIF	Regions of increased fluidity
PmB	Polymyxin B
PmE	Polymyxin E (colistin)
MDR	Multidrug–resistant
LPS	Lipopolysaccharide
FQs	Fluoroquinolones
ROS	Reactive oxygen species
QRDRs	Quinolone resistance–determining regions
RNAP	RNA polymerase
DC	Decoding center
PTC	Peptidyl transferase center
NPET	Nascent peptide exit tunnel
MAMs	Macrolide arrest motifs
AGAs	Aminoglycosides
2–DOS	2–deoxystreptamine
THF	Tetrahydrofolate
DHPS	Dihydropteroate synthase
DHFR	Dihydrofolate reductase
pABA	p–aminobenzoic acid
AI	Artificial intelligence
