# Supplementary material for: Antibiotic Mechanisms and Resistance: Molecular Insights and Therapeutic Strategies
Source: Antibiotics (Basel). 2026 Mar 29;15(4):351. doi: 10.3390/antibiotics15040351 (PMC13113030; doi:10.3390/antibiotics15040351)
Supplement: Supplementary file 1 [file antibiotics-15-00351-s001.zip › antibiotics-4161566-supplementary.pdf]

Supporting Information

Antibiotic Mechanisms and Resistance: Molecular Insights and Therapeutic Strategies

Haodi Ma[a†], Liying Zhang[a†], Lulu Wang[a,b], Zimeng Yang[a], Junfeng Liu[a], Haoyang Sun[a], Shuai Ge[a] , Chunshan Quan[a,b*]

[a]*Key Laboratory of Biotechnology and Bioresources Utilization (Ministry of Education), College of Life Science,Dalian Minzu University, 116600 Dalian, China*

[b]*Department of Bioengineering, College of Life Science, Dalian Minzu University, 116600, Dalian, China*

[*]Corresponding author: Chunshan Quan; E-mail address(es): mikyeken@dlnu.edu.cn.

[†]These authors made equal contributions to this work.

Table S1 Chemical structure and mechanism of action of antibiotics target cell wall

Category		Structural formula	Active site	Reference
**Fosfomycin**		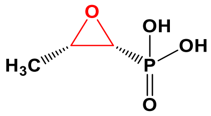	Forms a covalent bond with the active site of the MurA protein via its epoxide group (highlighted in red), thereby inhibiting the binding of PEP to MurA.	[1]
**Cyclosserine**	**D- cycloserine**	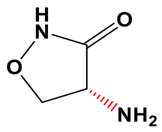	Inhibits D-alanyl-D-alanine synthetase, thereby preventing the synthesis of the D-Ala-D-Ala dipeptide. Additionally, inhibits D-alanyl-D-alanine ligase, which interferes with the cross-linking of peptidoglycan in the bacterial cell wall.	[2]
	**L-cycloserine**	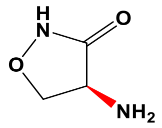	L-Cycloserine exhibits little or no antibacterial activity but can interact with neural pathways related to NMDA receptor signaling, producing effects on the nervous system	[3]
**Beta-lactam antibiotic**	**Penicillins**	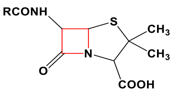	The β-lactam ring in the structure is the primary active site (highlighted in red). The opening of the β-lactam ring interacts with bacterial targets via acylation, thereby inhibiting bacterial growth.	[4,5]
	**Cephalosporins**	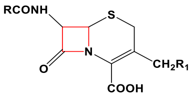		
	**Cephamycins**	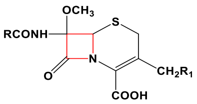		
	**Monocyclic β-lactams**	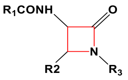		
	**Carbapenems**	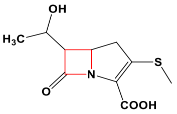		
**Bacitracin**		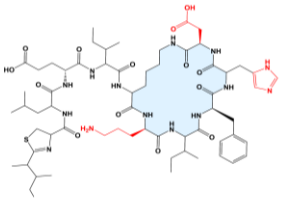	The antibacterial activity primarily depends on its cyclic peptide structure, where the D-Glu, D-Orn, and His residues (highlighted in red) serve as the key functional groups.	[6]
**Glycopeptide antibiotic (using Vancomycin as an example)**		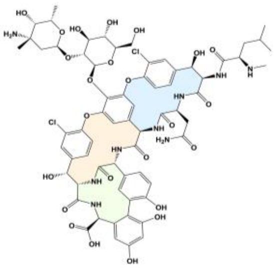	By virtue of its cyclic heptapeptide core structure, it forms hydrogen bonds with D-Ala-D-Ala, thereby inhibiting peptidoglycan synthesis in the bacterial cell wall.	[7]
**Isoniazid**		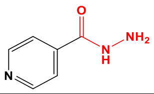	Relies on the acylhydrazone moiety (highlighted in red) within the molecular structure to exhibit antibacterial activity.	[8]
**Ethambutol**		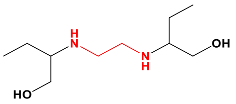	The diamine moiety within the molecule (highlighted in red) is capable of forming hydrogen bonds and electrostatic interactions with the active site of arabinosyltransferase.	[9]

Table S2 Chemical structures and mechanisms of action of antibiotics targeting the cell membrane

Category		Structural formula	Active site	Reference
**Daptomycin**		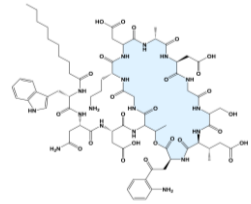	It inserts its lipophilic tail into the bacterial cell membrane and, with the assistance of Ca^{2+} ions interacting with membrane phospholipids, forms pores, disrupts ion homeostasis, and ultimately causes membrane destabilization. depolarization.	[10]
**Lantibiotics**	**Bliα**	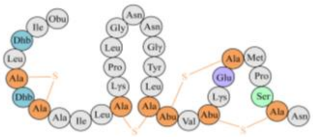	Bliα binds to lipid II upon interacting with the cell membrane, thereby disrupting membrane integrity. Meanwhile, Bli β forms pores within the membrane. This dual mechanism of action ultimately results in bacterial cell death.	[11]
	**Bliβ**	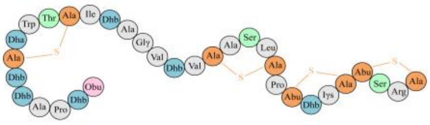		
**Polymyxin (using PmB as an example)**		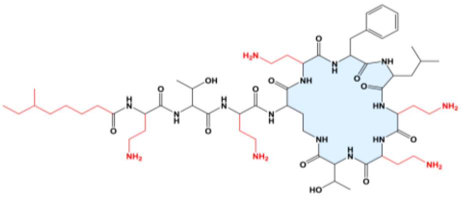	The multi-positively charged circular polypeptide structure in PmB specifically targets the bacterial outer membrane for binding and subsequently inserts its hydrophobic tail into the bacterial lipid bilayer, thereby disrupting membrane stability and permeability and ultimately leading to bacterial death.	[12]
**Short Peptides**	**Compound 1**	WRWRWR-NH2<br>RRRWWW-MH2<br>RWRWRW-NH2<br>……	The precise mechanism of action targeting the bacterial cell membrane requires further elucidation.	[13-15]
	**Compound 2**	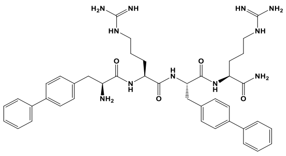		
	**Ltx5**	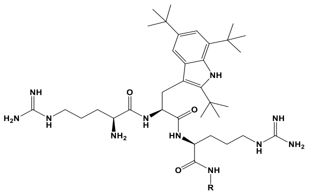		
**Other Compounds**	**Ceragenins (CAS-13)**	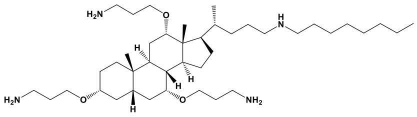	The precise mechanism of action targeting the bacterial cell membrane requires further elucidation.	[16]
	**Ceragenins (CAS-54)**	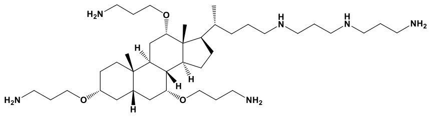		
	**Ceragenins (CAS-8)**	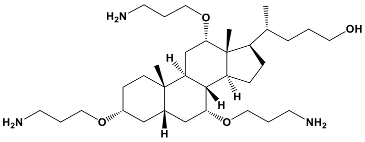		
	**Reutericyclin (DCAP)**	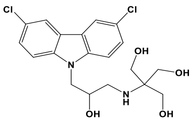		

Table S3 Chemical structures and mechanisms of action of antibiotics that inhibit nucleic acid synthesis

Category		Structural formula	Active site	Reference
**Quinolones**		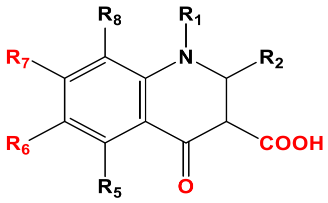	The core ring structure functions as a binding platform, enabling seamless integration into the DNA architecture. Moieties highlighted in red play a critical role in forming stable coordination bonds, thereby enhancing the overall molecular permeability.	[17,18]
**Metronidazole**		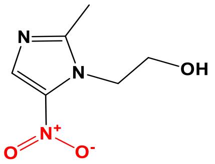	Under anaerobic conditions, the -NO_2 groups are reduced to generate reactive radicals, which subsequently cause disruption of the bacterial DNA structure.	[19]
**Nitrofurantoin**		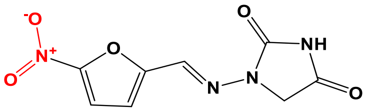	The -NO_2 groups within the structure are enzymatically reduced by bacteria to electrophilic intermediates, which subsequently exhibit antibacterial activity.	[20]
**Rifamycin**	**Rifampin**	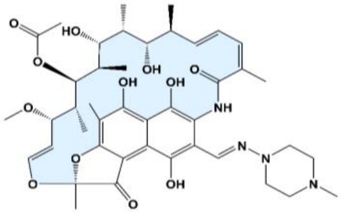	The blue-marked ring structure binds to the RNA polymerase and exerts its inhibitory effect by directly interacting with the enzyme's active site.	[21]
	**Rifapentine**	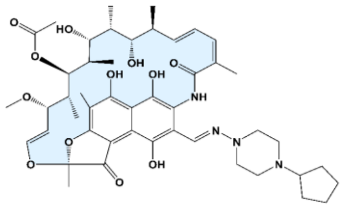		
	**Rifabutin**	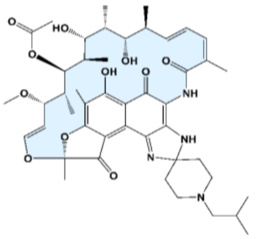		
	**Rifaximin**	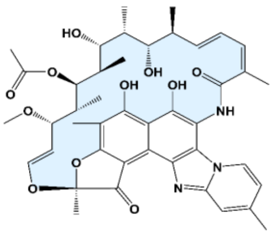		

Table S4 Chemical structures and mechanisms of action of antibiotics that inhibit protein synthesis

Category		Structural formula	Active site	Reference
Oxazolidinones	Linezolid	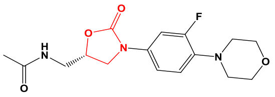	The site of action for the five-membered loop structure highlighted in red is PTC, which selectively binds to the A site within the 23S rRNA region of the 50S ribosomal subunit.	[22]
	Tedizolid	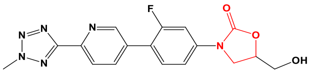		
Amphenicols (using Chloramphenicol as an example)		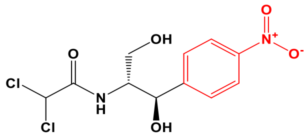	The nitrophenyl group highlighted in red plays a crucial role in ribosome binding, thereby determining the compound's antibacterial activity.	[23]
Macrolides	Erythromycin	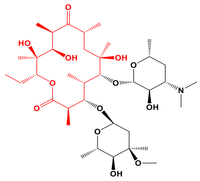	The macrolide lactone ring highlighted in red acts as the core structure, inserting into the NPET to inhibit peptide chain elongation.	[24]
	Ketolide (Telithromycin)	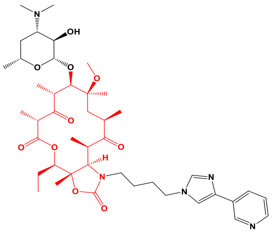		
Lincosamides (using Lincomycin as an example)		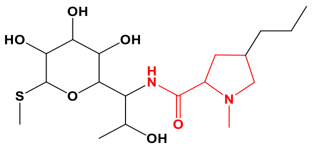	The pyrrolidine side chain induces significant steric hindrance within PTC and forms a hydrophobic interaction interface. The amide bond connects the two molecular domains, participates in hydrogen bonding with rRNA, and modulates the internal electronic distribution.	[25]
Tetracycline		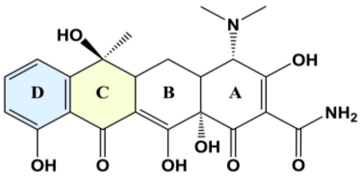	Their C and D rings induce steric hindrance with the first nucleotide of the anticodon in the tRNA bound to the A site. This steric interference spatially obstructs mRNA codon recognition by the aa-tRNA anticodon, thereby inhibiting peptide chain elongation and preventing bacterial replication.	[26]
Aminoglycoside	Streptomycin	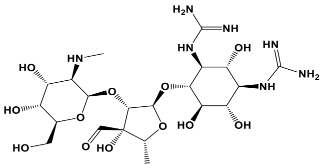	They bind to the 30S ribosomal subunit inside the bacterial cell, causing misreading of the mRNA and leading to incorrect translation.	[27]
	Apramycin	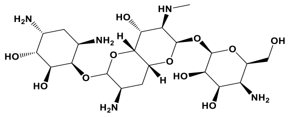		
	Neomycin	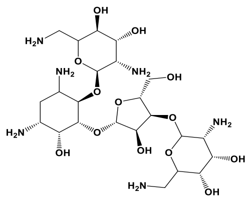		
	Gentamicin	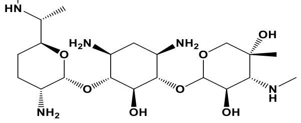		

References

1. Silver LL. Fosfomycin: Mechanism and Resistance. *Cold Spring Harb Perspect Med*. **2017**, 7, a025262. https://doi.org/10.1101/cshperspect.a025262.
2. Prosser GA.; de Carvalho LP. Reinterpreting the mechanism of inhibition of Mycobacterium tuberculosis D-alanine:D-alanine ligase by D-cycloserine. *Biochemistry*. **2013**, 52, 7145-7149. https://doi.org/10.1021/bi400839f.
3. Polc, P.; Pieri, L.; Bonetti, E. P.; Scherschlicht, R.; Moehler, H.; Kettler, R.; et al. L-cycloserine: behavioural and biochemical effects after single and repeated administration to mice, rats and cats. *Neuropharmacology*. **1986**, 25, 411-418. https://doi.org/10.1016/0028-3908(86)90236-4.
4. Mora-Ochomogo, M., & Lohans, C. T. β-Lactam antibiotic targets and resistance mechanisms: from covalent inhibitors to substrates. *RSC Med Chem*. **2021**, 12, 1623-1639. https://doi.org/10.1039/d1md00200g.
5. Hunashal, Y.; Kumar, G. S.; Choy, M. S.; D'Andréa, É. D.; Da Silva Santiago, A.; Schoenle, M. V.; et al. Molecular basis of β-lactam antibiotic resistance of ESKAPE bacterium E. faecium Penicillin Binding Protein PBP5. *Nat Commun*. **2023**, 14, 4268. https://doi.org/10.1038/s41467-023-39966-5.
6. Ciulla, M. G.; Gelain, F. Structure-activity relationships of antibacterial peptides. *Microb Biotechnol*. **2023**, 16, 757-777. https://doi.org/10.1111/1751-7915.14213.
7. Wang, F.; Zhou, H.; Olademehin, O. P.; Kim, S. J.; Tao, P. Insights into Key Interactions between Vancomycin and Bacterial Cell Wall Structures. *ACS Omega*. **2018**, 3, 37-45. https://doi.org/10.1021/acsomega.7b01483.
8. Sampiron, E. G.; Calsavara, L. L.; Baldin, V. P.; Montaholi, D. C.; Leme, A. L. D.; Namba, D. Y.; et al. Isoniazid-N-acylhydrazones as promising compounds for the anti-tuberculosis treatment. *Tuberculosis (Edinb)*. **2023**, 141, 102363. https://doi.org/10.1016/j.tube.2023.102363.
9. Zhang, L.; Zhao, Y.; Gao, Y.; Wu, L.; Gao, R.; Zhang, Q.; et al. Structures of cell wall arabinosyltransferases with the anti-tuberculosis drug ethambutol. *Science*. **2020**, 368, 1211-1219. https://doi.org/10.1126/science.aba9102.
10. Gray, D.A.; Wenzel, M. More than a pore: a current perspective on the in vivo mode of action of the lipopeptide antibiotic daptomycin. *Antibiotics*. **2020**, 9, 17. https://doi.org/10.3390/antibiotics9010017.
11. Barbosa, J.C.; Gonçalves, S.; Makowski, M.; Caetano, T.; Schneider, T.; et al. Insights into the mode of action of the two-peptide lantibiotic lichenicidin. *Colloids Surf B Biointerfaces*. **2022**;211:112308. https://doi.org/10.1016/j.colsurfb.2021.112308.
12. Kwa, A.; Kasiakou, S.K.; Tam, V.H.; Falagas, M.E. Polymyxin B: similarities to and differences from colistin (polymyxin E). Expert Rev. *Anti Infect. Ther*. **2007**, 5, 811–821. https://doi.org/10.1586/14787210.5.5.811.
13. Luo, X.; Wu, W.; Feng, L.; Treves, H.; Ren, M. Short peptides make a big difference: the role of botany-derived AMPs in disease control and protection of human health. *Int. J. Mol. Sci*. **2021**, 22, 11363. https://doi.org/10.3390/ijms222111363.
14. Strøm, M. B.; Haug, B. E.; Skar, M. L.; Stensen, W.; Stiberg, T.; Svendsen, J. S. The pharmacophore of short cationic antibacterial peptides. *J Med Chem*. **2003**, 46, 1567-1570. https://doi.org/10.1021/jm0340039.
15. Haug, B. E.; Stensen, W.; Kalaaji, M.; Rekdal, Ø.; Svendsen, J. S. Synthetic antimicrobial peptidomimetics with therapeutic potential. *J Med Chem*. **2008**, 51, 4306-4314. https://doi.org/10.1021/jm701600a.
16. Dias, C.; Rauter, A. P. Membrane-targeting antibiotics: recent developments outside the peptide space. *Future Med Chem*. **2019**, 11, 211-228. https://doi.org/10.4155/fmc-2018-0254.
17. Heeb, S.; Fletcher, M.P.; Chhabra, S.R.; Diggle, S.P.; Williams, P.; Cámara, M. Quinolones: from antibiotics to autoinducers. *FEMS Microbiol. Rev*. **2011**, 35, 247–274. https://doi.org/10.1111/j.1574-6976.2010.00247.x.
18. Bush, N.G.; Santos, I.; Abbott, L.R.; Maxwell, A. Quinolones: mechanism, lethality and their contributions to antibiotic resistance. *Molecules*. **2020**, 25, 5662. https://doi.org/10.3390/molecules25235662.
19. Dingsdag, S.A.; Hunter, N. Metronidazole: an update on metabolism, structure-cytotoxicity and resistance mechanisms. *J. Antimicrob. Chemother*. **2018**, 73, 265–279. https://doi.org/10.1093/jac/dkx351.
20. Wijma, R. A.; Fransen, F.; Muller, A. E.; Mouton, J. W. Optimizing dosing of nitrofurantoin from a PK/PD point of view: What do we need to know?. *Drug Resist Updat*. **2019**, 43, 1-9. https://doi.org/10.1016/j.drup.2019.03.001.
21. Adams, R. A.; Leon, G.; Miller, N. M.; Reyes, S. P.; Thantrong, C. H.; Thokkadam, A. M.; et al. Rifamycin antibiotics and the mechanisms of their failure. *J Antibiot*. **2021**, 74, 786-798. https://doi.org/10.1038/s41429-021-00462-x.
22. Leach, K.L.; Swaney, S.M.; Colca, J.R.; McDonald, W.G.; Blinn, J.R.; Thomasco, L.M.; et al. The site of action of oxazolidinone antibiotics in living bacteria and in human mitochondria. *Mol. Cell*. **2007**, 26, 393–402. https://doi.org/10.1016/j.molcel.2007.04.005.

23. Choi, J.; Marks, J.; Zhang, J.; Chen, D.H.; Wang, J.; Laslop, N.; et al. Dynamics of the context-specific translation arrest by chloramphenicol and linezolid. *Nat. Chem. Biol*. **2020**, 16, 310–317. https://doi.org/10.1038/s41589-019-0423-2.
24. Beckert, B.; Leroy, E.C.; Sothiselvam, S.; Bock, L.V.; Svetlov, M.S.; Graf, M.; et al. Structural and mechanistic basis for translation inhibition by macrolide and ketolide antibiotics. *Nat. Commun*. **2021**, 12, 4466. https://doi.org/10.1038/s41467-021-24674-9.
25. Schwarz, S.; Shen, J.; Kadlec, K.; Wang, Y.; Brenner Michael, G.; Fessler, A.T.; et al. Lincosamides, streptogramins, phenicols, and pleuromutilins: mode of action and mechanisms of resistance. Cold Spring Harb. *Perspect. Med*. **2016**, 6, a027037. https://doi.org/10.1101/cshperspect.a027037.
26. Chopra, I.; Roberts, M. Tetracycline antibiotics: mode of action, applications, molecular biology, and epidemiology of bacterial resistance. *Microbiol. Mol. Biol. Rev*. **2001**, 65, 232–260. https://doi.org/10.1128/MMBR.65.2.232-260.2001.
27. Kaufman, M.; Siomin, L.; Fridman, M. The relationship between the structure and toxicity of aminoglycoside antibiotics. *Bioorg. Med. Chem. Lett*. **2020**, 30, 127218. https://doi.org/10.1016/j.bmcl.2020.127218.
